# Control of Sleep by Dopaminergic Inputs to the *Drosophila* Mushroom Body

**DOI:** 10.3389/fncir.2015.00073

**Published:** 2015-11-09

**Authors:** Divya Sitaraman, Yoshinori Aso, Gerald M. Rubin, Michael N. Nitabach

**Affiliations:** ^1^Department of Cellular and Molecular Physiology, Yale University School of MedicineNew Haven, CT, USA; ^2^Janelia Research Campus, Howard Hughes Medical InstituteAshburn, VA, USA; ^3^Department of Genetics, Yale University School of MedicineNew Haven, CT, USA; ^4^Program in Cellular Neuroscience, Neurodegeneration and Repair, Yale University School of MedicineNew Haven, CT, USA

**Keywords:** sleep, *Drosophila melanogaster*, mushroom body, dopamine, synaptic plasticity

## Abstract

The *Drosophila* mushroom body (MB) is an associative learning network that is important for the control of sleep. We have recently identified particular intrinsic MB Kenyon cell (KC) classes that regulate sleep through synaptic activation of particular MB output neurons (MBONs) whose axons convey sleep control signals out of the MB to downstream target regions. Specifically, we found that sleep-promoting KCs increase sleep by preferentially activating cholinergic sleep-promoting MBONs, while wake-promoting KCs decrease sleep by preferentially activating glutamatergic wake-promoting MBONs. Here we use a combination of genetic and physiological approaches to identify wake-promoting dopaminergic neurons (DANs) that innervate the MB, and show that they activate wake-promoting MBONs. These studies reveal a dopaminergic sleep control mechanism that likely operates by modulation of KC-MBON microcircuits.

## Introduction

Parkinson's disease in human patients is strongly associated with disruption of sleep (Schrempf et al., 2014). Furthermore, the ascending dopamine system that projects from the brain stem to the forebrain and, specifically, to cerebral cortex is known to be important for regulating sleep and arousal, and is considered a wake-promoting pathway (Dzirasa et al., 2006; España and Scammell, 2011; Dauvilliers et al., 2015). However, despite this general knowledge concerning dopaminergic regulation of mammalian sleep through projections to cerebral cortex, underlying cellular, and molecular mechanisms remain poorly understood. In part, this is because of a lack of effective tools for highly restricted cell-specific experimental control of ascending dopaminergic systems in mammalian model systems such as rodents. We thus employed *Drosophila melanogaster* as a convenient experimentally tractable model system for studying cellular and molecular mechanisms of dopaminergic regulation of sleep.

The *Drosophila* mushroom body (MB) is an associative memory network of evolutionary origin likely conserved with mammalian cerebral cortex that has also been implicated in the control of sleep (Heisenberg et al., 1985; Zars et al., 2000; Joiner et al., 2006; Pitman et al., 2006; Yuan et al., 2006; Tomer et al., 2010; Guo et al., 2011; Seugnet et al., 2011; Yi et al., 2013; Ueno and Kume, 2014; Wu et al., 2014; Haynes et al., 2015). Fly sleep exhibits all of the key features of vertebrate sleep, including circadian regulation and homestatic rebound, and can be operationally identified as periods of locomotor quiescence lasting 5 min or longer (Hendricks et al., 2000; Shaw et al., 2000). The MB contains ~2000 KCs divided into 7 anatomical classes based on the specific projections of their axons into the α/β, α′/β′, or γ lobes (Tanaka et al., 2008). KCs receive synaptic inputs from sensory systems, such as olfactory projection neurons. KCs synapse convergently onto 34 MBONs of 21 cell types, whose dendrites contiguously tile the MB lobes, defining 15 non-overlapping compartments (Tanaka et al., 2008; Aso et al., 2014a). MBON cell types are uniquely named by their stereotyped arborizations in these compartments, numbered consecutively within each lobe starting from the intersection of the horizontal and vertical lobes (Aso et al., 2014a).

These 15 compartments are also each innervated by MB dopaminergic neurons (DANs). The axon terminals of ~130 DANs divided into 20 cell types tile the lobes isomorphically to the MBON dendrites (Aso et al., 2014a). While previous studies have implicated dopamine signaling (Kume et al., 2005) and several classes of DANs in the control of fly sleep (Shang et al., 2011; Liu et al., 2012b; Ueno et al., 2012), none of them innervate the MB, although it has recently been shown that manipulation of dopamine transporter expression using broadly expressed MB drivers influences sleep (Ueno and Kume, 2014).

To comprehensively identify the KCs, MBONs, and MB DANs that control sleep, we performed a thermogenetic neuronal activation screen using a new library of intersectional “split-GAL4” fly lines, each of which drives expression of an effector transgene under the control of the upstream activating sequence (UAS) to defined MB cell classes (Luan et al., 2006; Pfeiffer et al., 2010; Aso et al., 2014a,b; Sitaraman et al., 2015). We identified several classes of sleep-controlling MBONs with dendrites in distinct lobe compartments: cholinergic sleep-promoting MBON-γ2α′1, and glutamatergic wake-promoting MBON-γ5β′2a/β′2mp/β′2mp_bilateral and MBON-γ4 > γ1γ2 (Aso et al., 2014a,b). We also determined that α′/β′ and γm KCs are wake-promoting and γd KCs are sleep-promoting, and that α′/β′ and γm KCs promote wake by activating MBON-γ5β′2a/β′2mp/β′2mp_bilateral and γd KCs promote sleep by activating MBON-γ2α′1 (Sitaraman et al., 2015). Finally, we demonstrated that sleep deprivation activates γd KCs and MBON-γ2α′1 and activation of this synaptic microcircuit is required for homeostatic rebound sleep to occur after sleep deprivation (Sitaraman et al., 2015). Here we report the results of our screen of the MB DAN cell types, revealing that DANs projecting to lobe compartments containing the dendrites of wake-promoting MBONs are wake-promoting. We also demonstrate that activation of DANs projecting to the γ5 and β′2 compartments induces greater activation of MBON-γ5β′2a/β′2mp/β′2mp_bilateral than MBON-γ2α′1. These studies reveal a previously unknown dopaminergic sleep control mechanism that functions by modulation of the mushroom bodies, and provides for the first time a cellular and molecular substrate for control of sleep by dopaminergic modulation of an associative network.

## Experimental procedures

### Molecular and genetic methods

Genomic enhancers used in split-GAL4 and LexA transgenic lines were selected based on expression patterns of GAL4 lines using those same enhancers (Jenett et al., 2012) and constructed as previously described (Pfeiffer et al., 2010).

### Fly stocks

Flies were maintained on conventional cornmeal-agar-dextrose medium in 12 h light: 12 h dark conditions at 21°C (for dTRPA1 experiments) or 25°C (for immunohistochemistry), with ambient humidity of 60–70%. Flies were collected and tested for sleep 3–7 days after eclosion. *R58E02-LexA* is described in Jenett et al. (2012) and Liu et al. (2012a). Detailed genotype information of split-GAL4 strains—including p65ADZp-ZpGAL4DBD combinations—is as described (Aso et al., 2014a). p65ADZp fragments were inserted at attP40 or VK00027 while ZpGAL4DBD fragments were inserted at attP2. The studies reported here are exempt from ethical review, as they involve only *Drosophila* flies, and involve no vertebrate animals or human subjects. All MB split-GAL4 fly stocks can be obtained from Janelia Research Campus using the contact information at http://splitgal4.janelia.org/cgi-bin/splitgal4.cgi.

### Sleep assays

Split-GAL4 flies were crossed to flies bearing *10X UAS-dTRPA1* (in attP16) (Hamada et al., 2008) and maintained at 21–22°C. Genomic sites were chosen to avoid transvection, as described (Mellert and Truman, 2012). Male progeny, 3–7 days post-eclosion, were placed in 65 mm × 5 mm transparent plastic tubes with standard cornmeal dextrose agar media, placed in a Drosophila Activity Monitoring system (Trikinetics), and locomotor activity data were collected in 1 min bins. Activity monitors were maintained in a 12 h:12 h light-dark cycle at 65% relative humidity. Total 24-h sleep quantity (daytime plus nighttime sleep) was extracted from locomotor activity data as described (Donelson et al., 2012): sleep is defined as a contiguous period of inactivity lasting 5 min or more (Hendricks et al., 2000; Shaw et al., 2000). Sleep profiles were generated depicting average sleep (minutes per 30 min) for day 1 (baseline), days 2 and 3 (activation), and day 4 (recovery). In addition to permissive temperature controls other genotypic controls were used for hit detection as indicated. For all screen hits, waking activity was calculated as the number of beam crossings/min when the fly was awake. Temperature changes to activate and silence neurons are as indicated in **Figures 2**, **3**. Statistical comparisons between experimental and control genotypes were performed using Prism (Graphpad Inc, CA) by Kruskal-Wallis One-Way ANOVA followed by Dunn's *post-hoc* test or One-Way ANOVA followed by Dunnett's pairwise comparison test.

### Stimulation of PAM neurons by ATP/P2X2 and simultaneous GCamp6m imaging of MBONs

*LexAop2-dsRed* in attp18 (X); *LexAop2-P2X2* in su(Hw)attp5(II); *UAS-GCaMP6m* in VK0005(III) flies were generated using standard molecular and genetic methods, with the original transgenes as described (Pfeiffer et al., 2008, 2010; Yao et al., 2012; Chen et al., 2013). These flies were crossed to flies bearing appropriate LexA and split-GAL4 driver transgenes. To gain access to the PAM neurons for ATP application whole brain explants were placed on 8 mm diameter coverslips and placed in a recording chamber containing external solution (103 mM NaCl, 3 mM KCl, 5 mM N-tris (hydroxymethyl) methyl-2-aminoethane-sulfonic acid, 8 mM trehalose, 10 mM glucose, 26 mM NaHCO3, 1 mM NaH2PO4, 2 mM CaCl2, and 4 mM MgCl2, pH 7.4). For D1 dopamine receptor antagonist experiments, 100 μM SCH23390 was added to the external solution. The ATP ejection electrode was filled with freshly prepared 10 mM ATP solution and placed close to the PAM neurons using a micromanipulator. The PAM neurons were visually identified by their dsRed fluorescence. ATP was ejected by applying a 25 ms pressure pulse at 20 psi using a picospritzer (Parker Hannifin, Precision Fluidics Division, NH). The picospritizer was triggered by Zeiss image acquisition and processing software Zen pro 2012. Calcium imaging was performed with a Zeiss Axio Examiner Z1 upright microscope with W Plan Apochromat 40 × water immersion objective. GCaMP6m was excited with a 470 nm LED light source (Colibiri, Zeiss) and images were acquired using ORCA-R2 C10600-10B digital CCD camera (Hamamastu, Japan) at 3–10 Hz. For simultaneous imaging of spatially identical frames of dsRed (KCs) and GCaMP6m (MBONs) fluorescence we used a DV2 emission splitting system (Photometrics Inc). As a negative control, we imaged MBONs in brains of flies that lacked P2X2 expression in the PAM neurons and confirmed absence of Ca^2+^ increases in MBONs following ATP application (data not shown). The average fluorescence of all pixels for each time point in a defined ROI was subtracted from the average background fluorescence of an identically sized ROI elsewhere within the brain. The resulting pixel fluorescence value for each time point was defined as Ft. Changes in fluorescence were computed as %ΔF/Fo = ((Ft-Fo)/Fo) × 100, where Fo is defined as the average background-subtracted baseline fluorescence for the 10 frames preceding ATP application. All images were processed and quantified using Zen and Fiji (Image J).

### Immunohistochemistry

Dissection and immunohistochemistry of fly brains were performed as previously described with minor modifications (Jenett et al., 2012). Brains of 3–10 day old female flies were dissected in Schneider's Insect medium and fixed in 2% paraformaldehyde in Schneider's medium for 50 min at room temperature (RT). After washing in PBT (0.5% Triton X-100 in PBS), brains were blocked in 3% normal goat serum (or normal donkey serum, depending on the secondary antibody) for 90 min. Brains were then incubated in primary antibodies diluted in PBT for 2–4 days on a nutator at 4°C, washed three times in PBT for 30 min or longer, then incubated in secondary antibodies diluted in PBT for 2–4 days on a nutator at 4°C. Brains were washed thoroughly in PBT four times for 30 min or longer, and mounted in Vectashield (Vector laboratories, CA) for imaging. The following antibodies were used: anti-GFP (1:1000; Invitrogen; #AB124), mouse anti-nc82 (1:50; Developmental Studies Hybridoma Bank, Univ. Iowa), and cross-adsorbed secondary antibodies to IgG (H+L): goat Alexa Fluor 488 anti-rabbit (1:800; Invitrogen A11034) and goat Alexa Fluor 568 (1:400; Invitrogen A11031).

## Results

### Screen for MB DANs regulating sleep

**Figure 1:**
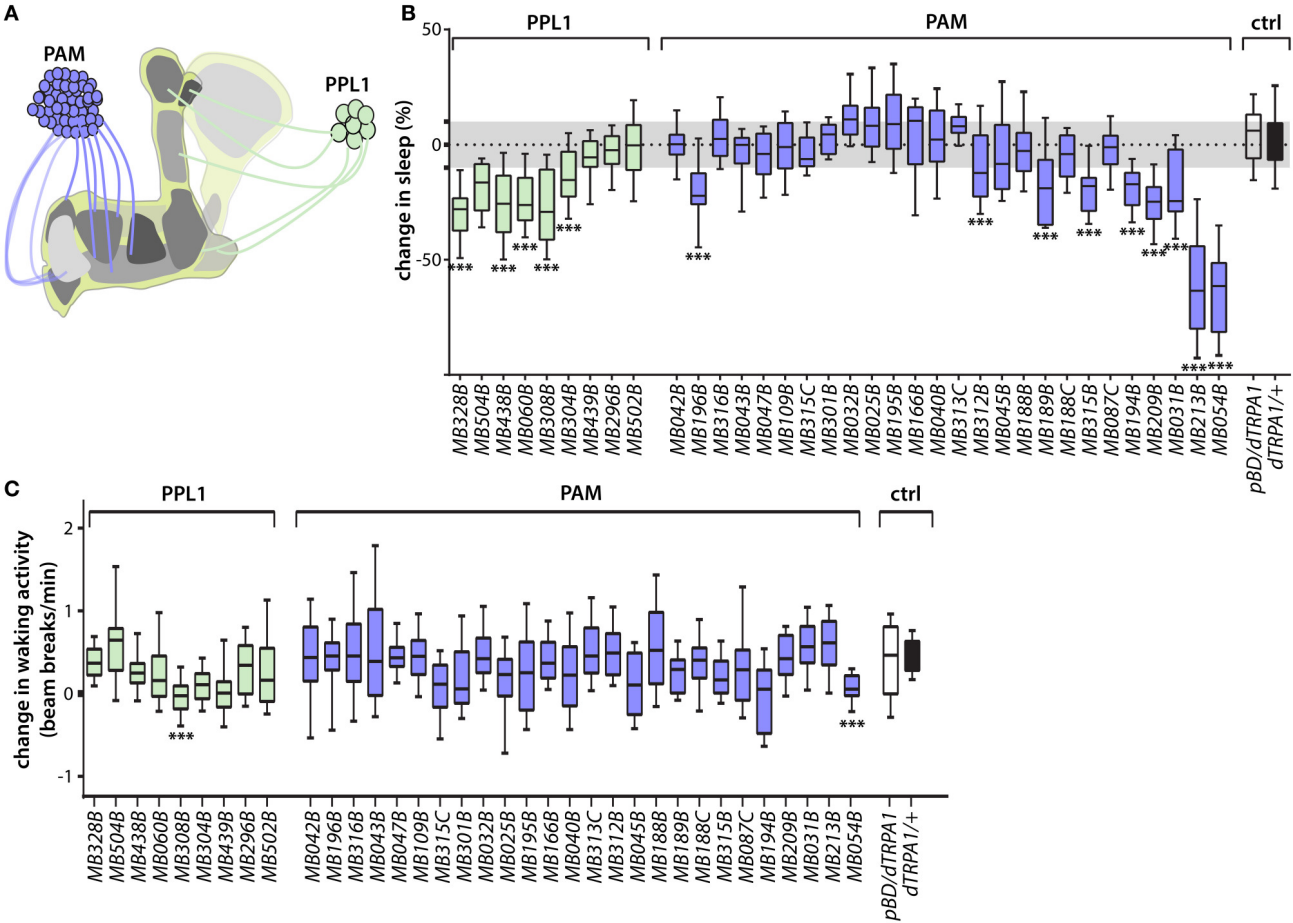
**Identification of wake-promoting MB dopamine neurons. (A)** Schematic representation of MB showing lobe-specific axon projections of PPL1 and PAM cluster dopamine neurons. **(B)** Change in sleep of male flies induced by activation of MB dopamine neuron subsets targeted by the indicated split-GAL4 driver lines to express dTRPA1 temperature-gated depolarizing cation channel. Percent change in sleep is defined as (sleep on day 1—sleep on day 2)/sleep on day 1, where the ambient temperature is increased on day 2 from 21.5°C to 28.5°C to activate the neurons expressing dTRPA1. Activation of various PAM and PPL1 dopamine neurons projecting to α′/β′ or γ lobes increases wake. Midline, box boundaries, and whiskers represent median, quartiles, and 10th and 90th percentiles, respectively. split-GAL4 lines are grouped by dopamine neuron cell cluster. Each split-GAL4 line was compared to an enhancerless GAL4 (*pBDPGAL4U*, indicated in the figures as *pBDG4U* or *pBD*) by Kruskal-Wallis non-parametric One-Way ANOVA and Dunn's *post-hoc* correction for multiple comparisons (^***^p < 0.001; *n* = 24–68 flies per genotype). **(C)** Change in waking locomotor activity of male flies induced by dTRPA1-mediated activation of DAN classes targeted by the indicated split-GAL4 driver lines. Change in activity is defined as the difference between average beam breaks/min during periods of wakefulness on day 2 (28.5°C) and day 1 (21.5°C) of the experiments. Strongly wake-promoting lines (*MB308B, MB054B*) exhibit significant decreases in waking locomotor activity. Midline, box boundaries, and whiskers represent median, quartiles, and 10th and 90th percentiles, respectively. Statistical comparison of each split-GAL4 line to an enhancerless GAL4 (*pBDG4U*) is by Kruskal-Wallis One-Way ANOVA and Dunnett's *post-hoc* correction for multiple comparisons (*n* = 24–68 flies for each genotype).

**Figure 2:**
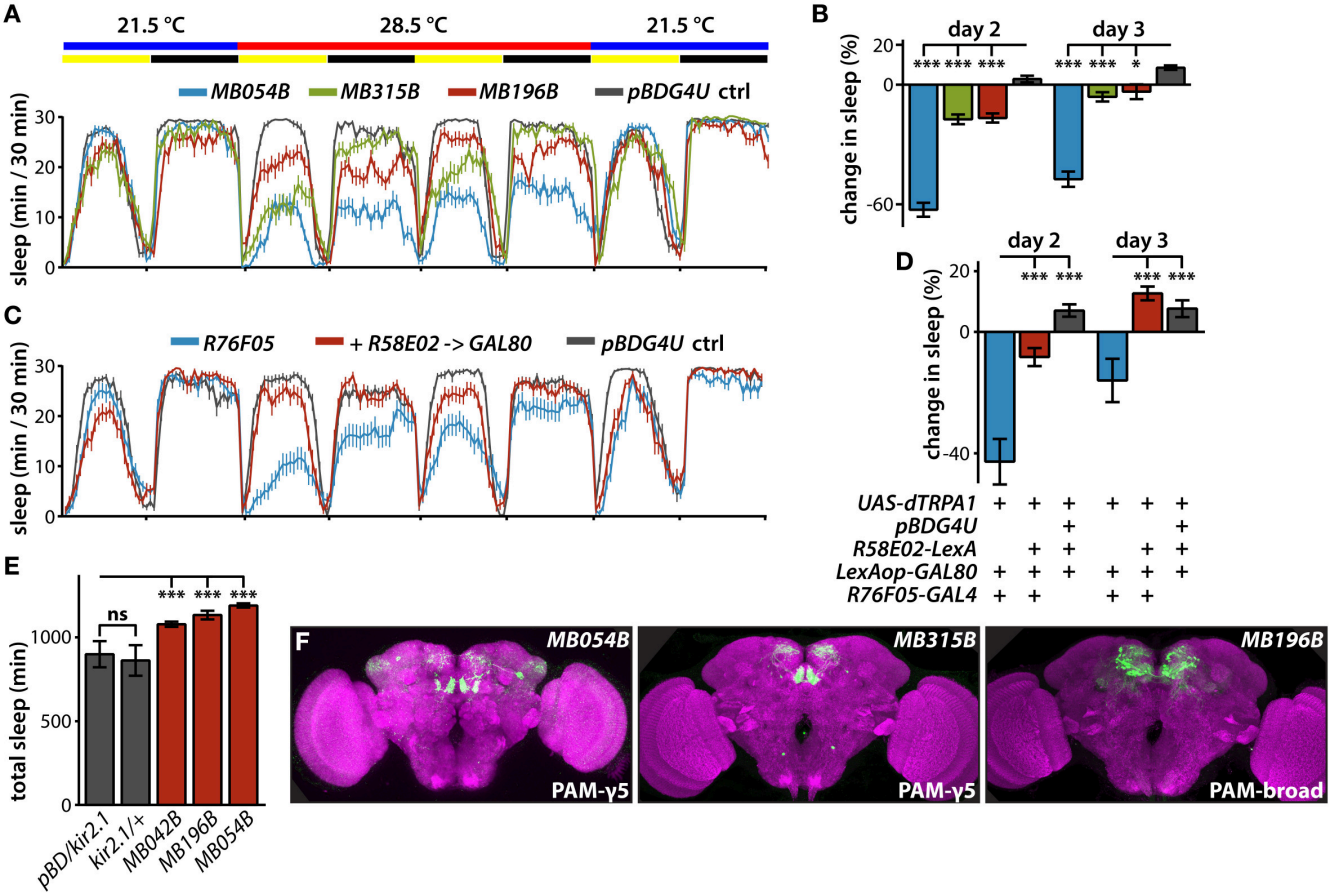
**Sleep profiles of flies following thermogenetic activation of MB dopamine neurons. (A)** Sleep profiles of male flies expressing dTRPA1 in PAM neurons driven by the indicated split-GAL4 lines (mean ± sem). Flies were maintained in 12 h:12 h light:dark (LD) conditions (indicated by the yellow and black bars, respectively), and the temperature was increased for days 2 and 3 of the 4 day experiment. DANs targeted by *MB054B* innervate the γ3 and γ5 compartments, *MB315B* the γ5 compartment, and *MB196B* multiple compartments. **(B)** Quantification of change in sleep of male flies expressing dTRPA1 in PAM neurons. Genotypes are color coded as in **(A)**. Statistical analysis was by One-Way ANOVA and Dunnett's paired-comparison test. **(C)** Sleep profiles of male flies expressing dTRPA1 in PAM neurons driven by *R76F05-GAL4* driver, with and without co-expression in PAM neurons of the GAL80 inhibitor of GAL4-mediated transcription driven by the *R58E02-LexA* driver, treated as in **(A)**. **(D)** Co-expression of GAL80 in PAM neurons strongly suppresses wake induced by GAL4-mediated expression of dTRPA1. Genotypes are color coded as in **(C)**; quantification and statistical analysis as in **(B)**; *n* = 26–29 flies per genotype. **(E)** Expression of the hyperpolarizing kir2.1 K^+^ channel driven in PAM neurons by the indicated split-GAL4 lines increases total 1-day sleep. Quantification and statistical analysis as in **(B)**; *n* = 30–32 flies per genotype. **(F)** Whole brains of flies expressing GFP under the control of indicated splitGAL4 drivers immunostained for GFP (green) and the synaptic neuropil marker BRUCHPILOT (purple). ^*^p < 0.05; ^***^p < 0.001.

The MB DANs are coarsely divided into PAM and PPL1 clusters based on the positions of their cell bodies (Figure 1A, Figure S1) (Budnik and White, 1988; Nassel and Elekes, 1992; Tanaka et al., 2008). Transient thermogenetic activation using the dTRPA1 temperature-gated depolarizing cation channel (Hamada et al., 2008) of some classes of PAM and PPL1 DANs suppresses sleep, in some cases very strongly (Figure 1B), and this effect is not attributable to non-specific effects on locomotion (Figure 1C). Sleep plots for MB DANs that innervate the γ5 compartment—which is also innervated by the dendrites of wake-promoting MBON-γ5β′2a/β′2mp/β′2mp_bilateral—are shown and quantified in Figures 2A,B. Lobe compartment innervation patterns of the DANs targeted by each split-GAL4 line are as previously described (Aso et al., 2014a; Yamagata et al., 2015), with images of selected lines shown in Figure 2F. Wake-promoting DANs project to various compartments in the γ and α′/β′ lobes, including γ5 and β′2, which contain dendrites of wake-promoting MBON-γ5β′2a/β′2mp/β′2mp_bilateral. None of the MB DANs increase sleep when activated, consistent with previous studies suggesting that dopamine is exclusively wake-promoting in flies (Liu et al., 2012b; Ueno et al., 2012; Ueno and Kume, 2014).

Expressing the GAL4 inhibitor, GAL80, with the *R58E02-LexA* driver suppresses the wake-promoting effect of dTRPA1-mediated activation using the *R76F05-GAL4* driver (Figures 2C,D), and these drivers overlap only in MB PAM neurons (Jenett et al., 2012). Furthermore, silencing neuronal activity of various PAM DANs by expression of the hyperpolarizing kir2.1 K^+^ channel (Baines et al., 2001; Nitabach et al., 2002) increases sleep (Figure 2E). Interestingly, while thermogenetic activation using the *MB042B* driver does not affect sleep, inactivation by kir2.1 expression using this driver increases sleep. While the reason for this is unclear, it could reflect different relative levels of GAL4-mediated transcriptional activation in the various PAM subsets targeted by this broad PAM driver. Regardless, these experiments further support a role for MB PAM neurons in the control of sleep.

**Figure 3:**
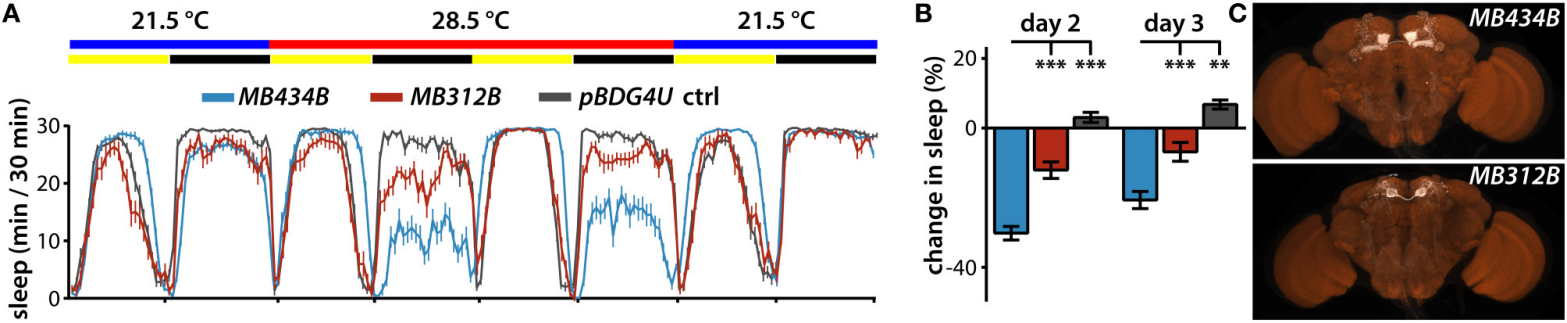
**Regulation of sleep by MBONs and DANs projecting to the γ4 compartment. (A)** Sleep profiles of male flies expressing dTRPA1 in MBON-γ4 > γ1γ2 using *MB434B* split-GAL4 driver (*n* = 32 flies) or in PAM DANs projecting to the γ4 compartment using *MB312B* (*n* = 31 flies). **(B)** Quantification of change in sleep of male flies expressing dTRPA1 in MBON-γ4 > γ1γ2 using *MB434B* split-GAL4 driver (*n* = 32 flies) or in PAM DANs projecting to the γ4 compartment using *MB312B* (*n* = 31 flies). Genotypes are color coded as in **(A)**. Statistical analysis was by One-Way ANOVA and Dunnett's paired-comparison test. **(C)** Whole brains of flies expressing GFP under the control of indicated splitGAL4 drivers immunostained for GFP (white) and the synaptic neuropil marker BRUCHPILOT (brown). ^**^p < 0.01; ^***^p < 0.001.

In addition to wake-promoting MBONs whose dendrites innervate γ5 and β′2 compartments, MBON-γ4>γ1γ2 is also wake-promoting (Aso et al., 2014b). Interestingly, transient activation of PAM neurons that project solely to the γ4 compartment containing dendrites of MBON-γ4 > γ1γ2 using the *MB312B* split-GAL4 suppresses sleep (Figures 3A–C). In some cases, thermogenetic activation using split-GAL4 lines targeting the same classes of MB DANs do not induce the same sleep phenotypes. For example, while *MB315B, MB315C*, and *MB313C* split-GAL4 lines each target PAM DANs projecting to the γ5 compartment, only *MB315B* suppresses sleep when used for thermogenetic activation (Figure 1B). Also, while *MB439B* and *MB296B* each target PPL1 DANs projecting to the γ2 and α′1 compartments innervated by sleep-promoting MBON-γ2α′1, thermogenetic activation using these split-GAL4 lines does not affect sleep (Figure 1B). These differences could be due to different levels of GAL4 expression in different split-GAL4 lines, and consequent differences in dTRPA1 expression, leading to differences in the degree of neural activation. Furthermore, there are some PPL1 lines that suppress sleep when used for thermogenetic activation (Figure 1B), while activation of the MBONs innervating the same compartments does not (Aso et al., 2014b). This suggests that strong thermogenetic activation could induce spillover of dopamine across compartments (which are not isolated from one another by glia), while endogenous dopamine secretion could be weaker and thus more specific. Future studies following up on additional specific sleep-regulating MB DAN classes is likely to resolve this question raised by the comprehensive screen we report here.

### MB DANs activate wake-promoting MBONs

**Figure 4:**
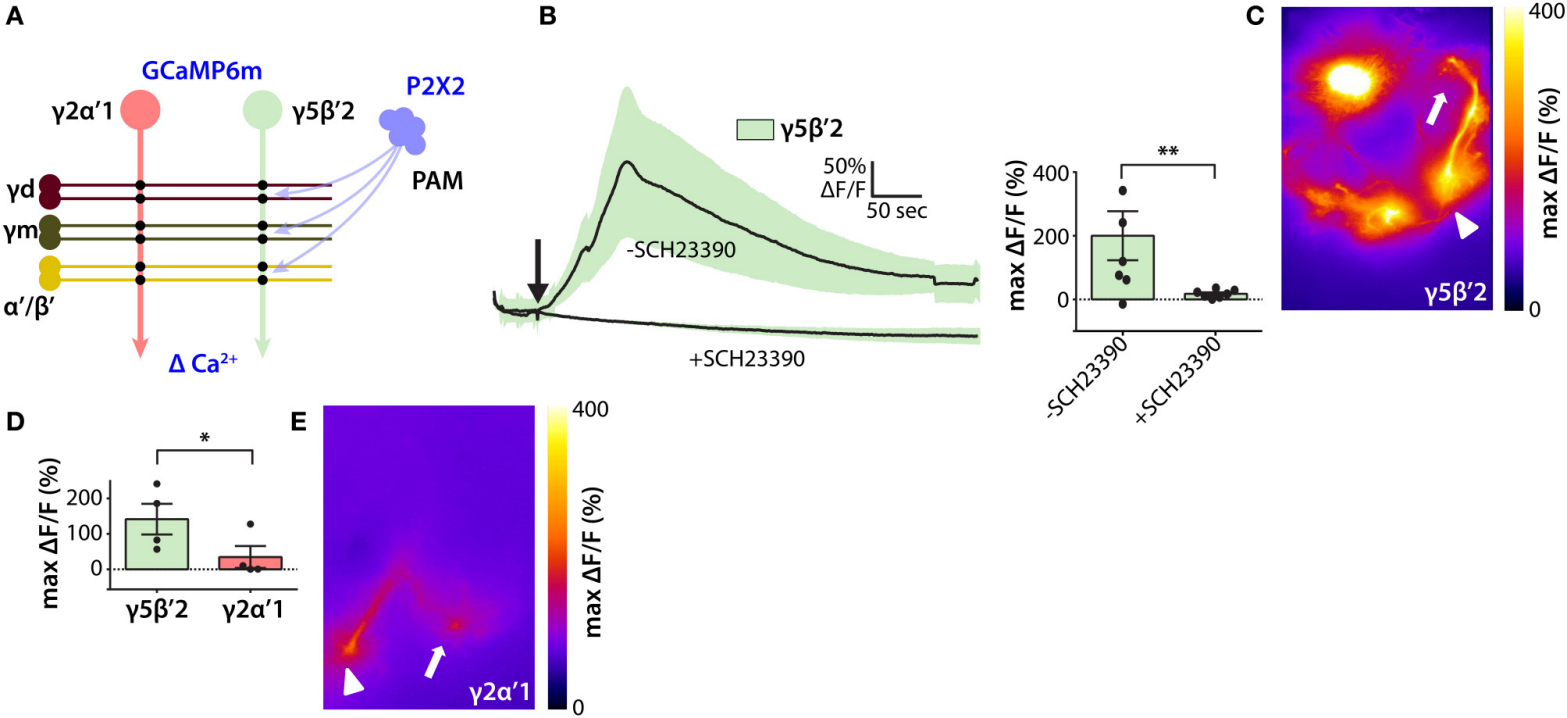
**Functional inputs of PAM dopamine neurons to the wake-promoting MB microcircuit. (A)** Schematic of experimental design to probe functional connections between wake-promoting PAM neurons and sleep-regulating MBONs. P2X2 ATP-gated depolarizing cation channel was expressed in PAM dopamine neurons, and GCaMP6m fluorescent Ca^2+^ indicator in MBONs. **(B)** Ca^2+^ responses of GCaMP6m-expressing γ5β′2-MBONs following ATP-mediated activation of P2X2 expressed in PAM neurons using *R58E02-LexA*. Line graph depicts ΔF/F time course in the presence and absence of dopamine receptor D1 subtype blocker SCH23390 (mean ± sem); bar graph depicts peak ΔF/F (mean ± sem). Statistical comparison of peak responses was by unpaired *t*-test (*n* = 6 brains per condition). **(C)** Heat map showing peak Ca^2+^ response of representative GCaMP6m-expressing γ5β′2-MBONs following ATP-mediated PAM activation. Arrowhead indicates MBON dendrites and arrow indicates axon terminals; panel width is 50 μm. **(D)** Peak Ca^2+^ responses of GCaMP6m-expressing γ2α′1 and γ5β′2-MBONs after ATP-mediated activation of P2X2-expressing PAM neurons. γ5β′2-MBONs respond more strongly than γ2α′1 to PAM activation. Quantification and statistical analysis as in **(B)** (*n* = 4 brains per genotype). **(E)** Heat map showing peak Ca^2+^ response of GCaMP6m-expressing MBON-γ2α′1 following ATP-mediated PAM activation. Symbols as in **(C)**; panel width is 50 μm. ^*^p < 0.05; ^**^p < 0.01.

To test the hypothesis that MB DANs promote wake by increasing activation of the wake-promoting γ5β′2 microcircuit, we activated P2X2-expressing MB DANs and measured Ca^2+^ responses in MBON-γ5β′2a/β′2mp/β′2mp_bilateral (Figure 4A). Because of the lack of available LexA drivers specific for DANs projecting to γ5 or β′2, we drove P2X2 expression using the relatively broad *R58E02-LexA* PAM driver that projects to the γ5 and β′2 compartments, among others, but is not active in non-PAM neurons (Liu et al., 2012a). Consistent with our hypothesis, transient activation of PAM DANs induces robust transient Ca^2+^ increases in the dendrites of MBON-γ5β′2a/β′2mp/β′2mp_bilateral in both the γ5 and β′2 compartments (Figures 4B,C). Complete block by bath application of the competitive D1-specific dopamine receptor antagonist SCH23390 at 100 μM (Boto et al., 2014) establishes the underlying molecular basis for this response, and confirms that it is mediated by dopamine acting on D1 receptors (Figure 4B).

Since PAM DANs do not project to the γ2 or α′1 compartments (Aso et al., 2014a) we assessed the compartmental specificity of dopamine secretion into the MB by comparing Ca^2+^ responses of MBON-γ5β′2a/β′2mp/β′2mp_bilateral and MBON-γ2α′1 to PAM DAN activation. Consistent with compartmental specificity, transient activation of PAM DANs induces significantly greater Ca^2+^ increases in MBON-γ5β′2a/β′2mp/β′2mp_bilateral than in MBON-γ2α′1 (Figures 4D,E). Taken together, our results support the hypothesis that MB DANs promote wake by activating γ5β′2 and γ4 > γ1γ2 wake-promoting MB microcircuits. Note, however, that our results do not exclude an indirect pathway whereby dopamine secreted by sleep-regulating MB DANs activates sleep-regulating MBONs by activating D1 receptors in other neurons presynaptic to MBONs, specifically KCs. Future studies, such as the suppression of D1 receptor expression specifically in MBONs using UAS-RNAi hairpin transgenes, are likely to resolve this question.

## Discussion

We have used a combination of sophisticated cell-specific genetic manipulations with behavioral sleep analysis and optical electrophysiology to identify compartment-specific wake-promoting MB DANs that activate wake-promoting microcircuits. Previous studies have implicated DANs innervating the central complex (CX)—a brain region involved in locomotor control (Strauss and Heisenberg, 1993)—in regulating sleep (Liu et al., 2012b; Ueno et al., 2012), and other non-dopamingeric CX neurons have been implicated in homeostatic control of sleep (Donlea et al., 2011, 2014). In addition, it has recently been shown that manipulations of dopamine signaling in the MB alter sleep, although the precise DANs involved remains unclear (Ueno and Kume, 2014). We have now identified specific wake-promoting MB DANs and shown that they innervate lobe compartments also innervated by wake-promoting KCs and MBONs (Figures 1–3). Importantly, we have also shown that dopamine secretion by DANs innervating a particular MB lobe compartment acts through D1 subtype receptors to activate the wake-promoting microcircuit specific to that compartment to a much greater extent than it activates the sleep-promoting microcircuit residing in different compartments (Figure 4). This provides direct physiological evidence for compartment-specific dopamine signaling in the regulation of sleep by the MB, and is consistent with a previous study in the context of learning and memory (Boto et al., 2014). Future studies are required to determine additional cellular and molecular details of how dopamine signals modulate sleep-regulating microcircuits.

On the basis of recently published studies of MB control of sleep and the results presented here, we propose a unified mechanistic model for homeostatic control of sleep by excitatory microcircuits in the *Drosophila* MB. Wake-promoting MBON-γ5β′2a/β′2mp/β′2mp_bilateral and sleep-promoting MBON-γ2α′1 each receive anatomical inputs from both wake-promoting γm and α′/β′ KCs and sleep-promoting γd KCs (Aso et al., 2014a). However, segregation of sleep control information into separate microcircuits is enforced by greater synaptic weights between γm and α′/β′ KCs and MBON-γ5β′2a/β′2mp/β′2mp_bilateral, and between γd KCs and MBON-γ2α′1 (Sitaraman et al., 2015). We thus hypothesize that compartment-specific dopamine signals from MB DANs could potentially determine these differences in synaptic weight. Future studies will test this hypothesis.

Interestingly, other fly behaviors have recently been found to be regulated by sleep-controlling compartment-specific MB microcircuits. For example, the integration of food odor to suppress innate avoidance of CO_2_ is mediated by MBON-γ5β′2a/β′2mp/β′2mp_bilateral and PAM DANs that innervate the β′2 compartment (Lewis et al., 2015). Optogenetic activation experiments reveal that wake-promoting MBON-γ5β′2a/β′2mp/β′2mp_bilateral mediates innate avoidance, while MBON-γ2α′1 mediates attraction (Aso et al., 2014b). However, thermogenetic inactivation studies reveal that both MBON-γ5β′2a/β′2mp/β′2mp_bilateral and MBON-γ2α′1 are important for various forms of associative memory formation (Aso et al., 2014b). These diverse waking behaviors that involve the activity of sleep-regulating neurons raises the interesting question whether such roles are independent, or causally linked, which future studies can address.

Importantly, we have provided for the first time a cellular and molecular mechanism for for dopaminergic control of sleep through modulation of an associative network. While dopaminergic projections to cerebral cortex are known to be important for regulating sleep and arousal in mammals, underlying cellular and molecular mechanisms remain poorly understood (España and Scammell, 2011; Dauvilliers et al., 2015), although D2 subtype dopamine receptors have been implicated in the control of REM sleep (Dzirasa et al., 2006). Because of the possible evolutionary relationship between the MB and vertebrate forebrain associative networks (such as mammalian cerebral cortex), these studies thus provide a framework for the design of analogous experiments in genetically tractable vertebrate model systems such as zebrafish and mice.

## Author contributions

Design of experiments: DS, YA, GR, MN. Performing experiments: DS, YA. Analyzing data: DS, YA. Drafting manuscript: DS, YA, GR, MN.

### Conflict of interest statement

The authors declare that the research was conducted in the absence of any commercial or financial relationships that could be construed as a potential conflict of interest.

## Supplementary material

Figure S1 **Lobe compartment innervation pattern of all screened MB DAN split-GAL4 lines**.
