# Corrigendum: Control of Sleep by Dopaminergic Inputs to the *Drosophila* Mushroom Body

**DOI:** 10.3389/fncir.2015.00084

**Published:** 2015-12-22

**Authors:** Divya Sitaraman, Yoshinori Aso, Gerald M. Rubin, Michael N. Nitabach

**Affiliations:** ^1^Department of Cellular and Molecular Physiology, Yale University School of MedicineNew Haven, CT, USA; ^2^Janelia Research Campus, Howard Hughes Medical InstituteAshburn, VA, USA; ^3^Department of Genetics, Yale University School of MedicineNew Haven, CT, USA; ^4^Program in Cellular Neuroscience, Neurodegeneration and Repair, Yale University School of MedicineNew Haven, CT, USA

**Keywords:** sleep, *Drosophila melanogaster*, mushroom body, dopamine, synaptic transmission

The citation for the first use of P2X2 as an exogenous chemogenetic neural activator (Lima and Miesenbock, 2005) was inadvertently omitted from the manuscript on page 2, at the end of the first sentence of the Section entitled “Stimulation of PAM Neurons by ATP/P2X and Simultaneous GCamp6m Imaging of MBONs.”

## Conflict of interest statement

The authors declare that the research was conducted in the absence of any commercial or financial relationships that could be construed as a potential conflict of interest.
